# Some Cases of Neuralgia

**Published:** 1829-10-01

**Authors:** 


### SOME CASES OF NEURALGIA. By Dr. PASSAGAY.

*Case* 1.—GASTRIC NEURALGIA CONVERTED INTO DENTAL NEURALGIA.

Miss Arnaud, aged 33, enjoyed good health habitually, with the exception of attacks of tooth-ache, by which she had lost some of her teeth. She was suddenly seized, in the middle of Autumn, with a species of gastrodynia, severe pain in the epigastrium, flatulence, weight, eructation, nausea and vomiting of aliments, constipation. She now remembered that, in Autumn, she had before been frequently affected with slight dyspepsia, occasioned, as she thought, by eating too much fruit. For seven months after this last attack, she was unable to bear more than a few spoonfuls of food at a time, without experiencing dreadful sufferings. Two physicians, whom she had consulted, declared that she laboured under scirrhus of the pylorus, and prescribed accordingly. During three months that she followed their advice, her sufferings became greatly aggravated. The pain, on taking the smallest quantity of food, was not confined to the region of the stomach, but occupied the superior dental arch, where it was intolerable. In this state she went on some months more, and was now wasting away with a hectic fever, vertigos, faintings, &c. so that her death appeared fast approaching. Seven physicians were called into consultation, and retired without deciding on any thing useful. The author was then left alone; and having viewed the case as one of neuralgia rather than of cancer, he entertained some hopes of recovery. He prescribed opium combined with aloetic aperients, and stimulant frictions, with anodynes to the region of the stomach. Nutritious lavements were daily thrown up, and gentle carriage exercise recommended. In one month of this plan she was restored to health.

*Case* 2.—CHRONIC NEURALGIA OF THE STOMACH, AFTERWARDS OF THE ILIUM.

A youth, 16 years of age, came under our author's care on the 20th December. He was pale and emaciated with long suffering. Every evening, between five and seven o'clock, he was seized with an intense pain in the region of the stomach, accompanied by nausea, vomiting and obstinate constipation. The accession lasted from six to eight hours, and was never over before midnight. This state had continued, with little or no variation, for six years. The appetite and sleep were little affected, and the other functions were undisturbed. One circumstance was remarkable. The disease ceased for about a month every Spring, and then returned as bad as ever. When the boy was brought to our author, the seat of the pain had changed from the epigastrium to the right iliac region, which was tender on pressure. In all other respects, the complaint was precisely the same. The pain came on every evening at seven o'clock. Every kind of antiphlogistic remedy and depletion had been unsparingly used, without the least success. In one week the malady ceded to stimulant frictions—antispasmodics—and a combination of sulphate of quinine with subnitrate of bismuth and calcined magnesia. Six years have now elapsed without any return of the disease.—CLINIQUE.

---

